# Supplementary material for: Underwater hyperspectral classification of deep sea corals exposed to 2-methylnaphthalene
Source: PLoS One. 2019 Feb 27;14(2):e0209960. doi: 10.1371/journal.pone.0209960 (PMC6392237; doi:10.1371/journal.pone.0209960)
Supplement: S1 Table — shows the concentration of 2-methylnaphthalene measured in water samples collected from stock solutions every 24 h during the 96 h exposure period. The average (avg) value and corresponding standard deviation (std dev) are also given. Nominal concentrations, Cnom, are listed for each treatment group. Each water sample was performed in duplicate, with each duplicate labeled I or II in the table. Regarding the analyses performed on the 2-methylnaphthalene water samples, the limit of quantification is 0.1 mg L−1, and the uncertainty is 20% at the limit of quantification. This uncertainty decreases above the limit of quantification. (PDF) [file pone.0209960.s001.pdf]

S1 Table. Measured 2-methylnaphthalene concentration $(\mathrm{mg\,L^{-1}})$ **in stock bottles**

Treatment group	C0			C1			C2			C3			C4		
C_{nom} $(\mathrm{mg\,L^{-1}})$	0.00			1.03			2.27			5.00			>8.00		
Time (h) \ Replicate	I	II	avg	I	II	avg	I	II	avg	I	II	avg	I	II	avg
0	<LOD	<LOD	<LOD	1.4	1.3	1.3	3.2	3.2	3.2	3.0	3.0	3.0	7.9		7.9
24 B	<LOD	<LOD	<LOD	1.2	1.2	1.2	3.3	3.2	3.2	5.2	5.0	5.1	8.3	9.6	9.0
proportion			-			89			101			170			113
24 A	<LOD	<LOD	<LOD	1.5	1.5	1.5	2.9	2.8	2.8	1.8	1.8	1.8	7.8	8.2	8.0
48 B	<LOD	<LOD	<LOD	1.5	1.5	1.5	3.1	3.0	3.1	4.4	4.5	4.4	8.2	7.8	8.0
proportion			-			104			109			251			100
48 A	<LOD	<LOD	<LOD	1.3	1.3	1.3	2.4	2.5	2.4	5.0	4.4	4.7	10.2	7.3	8.8
72 B	<LOD	<LOD	<LOD	1.3	1.0	1.2	2.6	2.7	2.6	4.3	4.7	4.5	9.8	8.0	8.9
proportion			-			92			108			95			101
72 A	<LOD	<LOD	<LOD	1.1	1.0	1.1	2.1	1.9	2.0	4.7	4.3	4.5	7.4	6.1	6.7
96 B	<LOD	<LOD	<LOD	1.1	1.1	1.1	2.4	2.5	2.4	4.8	4.9	4.9	8.1	8.0	8.1
proportion			-			102			123			107			120
Avg concentration	<LOD	<LOD	<LOD	1.3	1.2	1.3	2.7	2.7	2.7	4.2	4.1	4.1	8.5	7.9	8.2
St dev concentration	<LOD	<LOD	<LOD	0.2	0.2	0.2	0.4	0.5	0.4	1.2	1.1	1.1	1.0	1.1	0.7
% of nominal			-	126	120	123	121	119	120	83	81	82	106	98	102
Avg proportion			-			97			110			156			109

B refers to "Before Stock bottle replacement" meaning samples collected when the volume left in the stock bottles were at a minimum of approximately 4 L. A refers to "After Stock bottle replacement" meaning samples collected when the volume in the stock bottles were at its maximum of 20 L. At $t = 96$ h water samples were collected when the volume in the stock bottle used from $t = 72$ h was at a minimum. By proportion we refer to the concentration at the end compared to the start of the 24 h cycle where the same stock solvent was used, and reflect the change in concentration over time. The bottom four rows indicate average, standard deviation percentage of nominal concentration and average proportion for each column, respectively. The missing value in column C4 II was not analyzed. The limit of detection (LOD) was 0.033mg $\mathrm{L^{-1}}$.

S1 Table.
