# Supplementary material for: Underwater hyperspectral classification of deep sea corals exposed to 2-methylnaphthalene
Source: PLoS One. 2019 Feb 27;14(2):e0209960. doi: 10.1371/journal.pone.0209960 (PMC6392237; doi:10.1371/journal.pone.0209960)
Supplement: S2 Table — shows the concentration of 2-methylnaphthalene for the exposure beakers. The exposure was performed with 3 corals in each exposure beaker, across 4 replicates for each of the 5 treatment groups. Regarding the analyses performed on the 2-methylnaphthalene water samples, the limit of quantification is 0.1 mg L−1, and the uncertainty is 20% at the limit of quantification. This uncertainty decreases above the limit of quantification. Additional uncertainty is expected as the magnetic stirring mechanism cannot operate at high frequency, as this would disturb the coral samples. (PDF) [file pone.0209960.s002.pdf]

S2 Table. Measured 2-methylnaphthalene concentration $(\mathrm{mg\,L^{-1}})$ in exposure beakers

Treatment group		C0			C1			C2			C3			C4		
	C_{nom} $(\mathrm{mg\,L^{-1}})$	0.00			1.03			2.27			5.00			8.00		
Time (h)	Replicate	I	II	avg	I	II	avg	I	II	avg	I	II	avg	I	II	avg
0	R1	0.0	0.0	0.0	0.0	0.0	0.0	0.4	0.4	0.4	0.4	0.4	0.4	1.1	1.0	1.1
	R2	0.0	0.0	0.0	0.1	0.1	0.1	0.4	0.4	0.4	0.0	0.0	0.0	0.0	0.0	0.0
	R3	0.0	0.0	0.0	0.1	0.1	0.1	0.0	0.0	0.0	0.4	0.4	0.4	1.0	1.0	1.0
	R4	0.1	0.0	0.0	0.1	0.1	0.1	0.0	0.0	0.0	0.4	0.4	0.4	0.0	0.0	0.0
24	R1	0.0	0.0	0.0	0.4	0.4	0.4	1.9	2.0	2.0	1.1	1.3	1.2	2.0	2.5	2.2
	R2	0.0	0.0	0.0	0.4	0.4	0.4	0.9	1.1	1.0	0.0	0.0	0.0	2.2	2.8	2.5
	R3	0.0	0.0	0.0	0.4	0.5	0.5	1.2	1.4	1.3	0.6	1.4	1.0	3.2	3.8	3.5
	R4	0.0	0.0	0.0		0.8	0.8	1.1	1.1	1.1	0.7	1.4	1.1	1.6	2.6	2.1
48	R1	0.0	0.0	0.0	0.6	0.6	0.6	1.8	1.9	1.9	2.0	2.0	2.0	3.3	3.9	3.6
	R2	0.0	0.0	0.0	0.6	0.7	0.6	1.2	1.3	1.3	0.5	0.0	0.3	3.4	3.9	3.7
	R3	0.0	0.0	0.0	0.7	0.7	0.7	1.4	1.5	1.5	2.7	2.0	2.3	4.1	4.9	4.5
	R4	0.0	0.0	0.0	0.7	0.7	0.7	1.3	1.4	1.3	1.3	2.1	1.7	2.6	3.2	2.9
72	R1	0.0	0.0	0.0	0.6	0.6	0.6	1.2	1.3	1.2	2.1	2.3	2.2	3.2	3.9	3.5
	R2	0.0	0.0	0.0	0.6	0.7	0.6	1.1	1.2	1.2	0.0	0.0	0.0	2.3	4.2	3.3
	R3	0.0	0.0	0.0	0.3	0.3	0.3	1.3	1.4	1.3	8.9	2.4	5.7	4.2	4.6	4.4
	R4	0.0	0.0	0.0	0.6	0.7	0.7	1.2	1.4	1.3	2.7	2.4	2.6	3.0	3.5	3.2
	avg	0.0	0.0	0.0	0.4	0.5	0.5	1.0	1.1	1.1	1.5	1.2	1.3	2.3	2.9	2.6
	std dev	0.0	0.0	0.0	0.2	0.3	0.3	0.6	0.6	0.6	2.2	1.0	1.5	1.3	1.6	1.4
	% of nominal						44			47			27			32

The bottom three rows indicate average, standard deviation, and percentage of nominal concentration for each column, respectively. The limit of detection (LOD) was $0.033\mathrm{mg\,L^{-1}}$.

S2 Table.
