# Supplementary figures and images for: Underwater hyperspectral classification of deep sea corals exposed to 2-methylnaphthalene
Source: PLoS One. 2019 Feb 27;14(2):e0209960. doi: 10.1371/journal.pone.0209960 (PMC6392237; doi:10.1371/journal.pone.0209960)

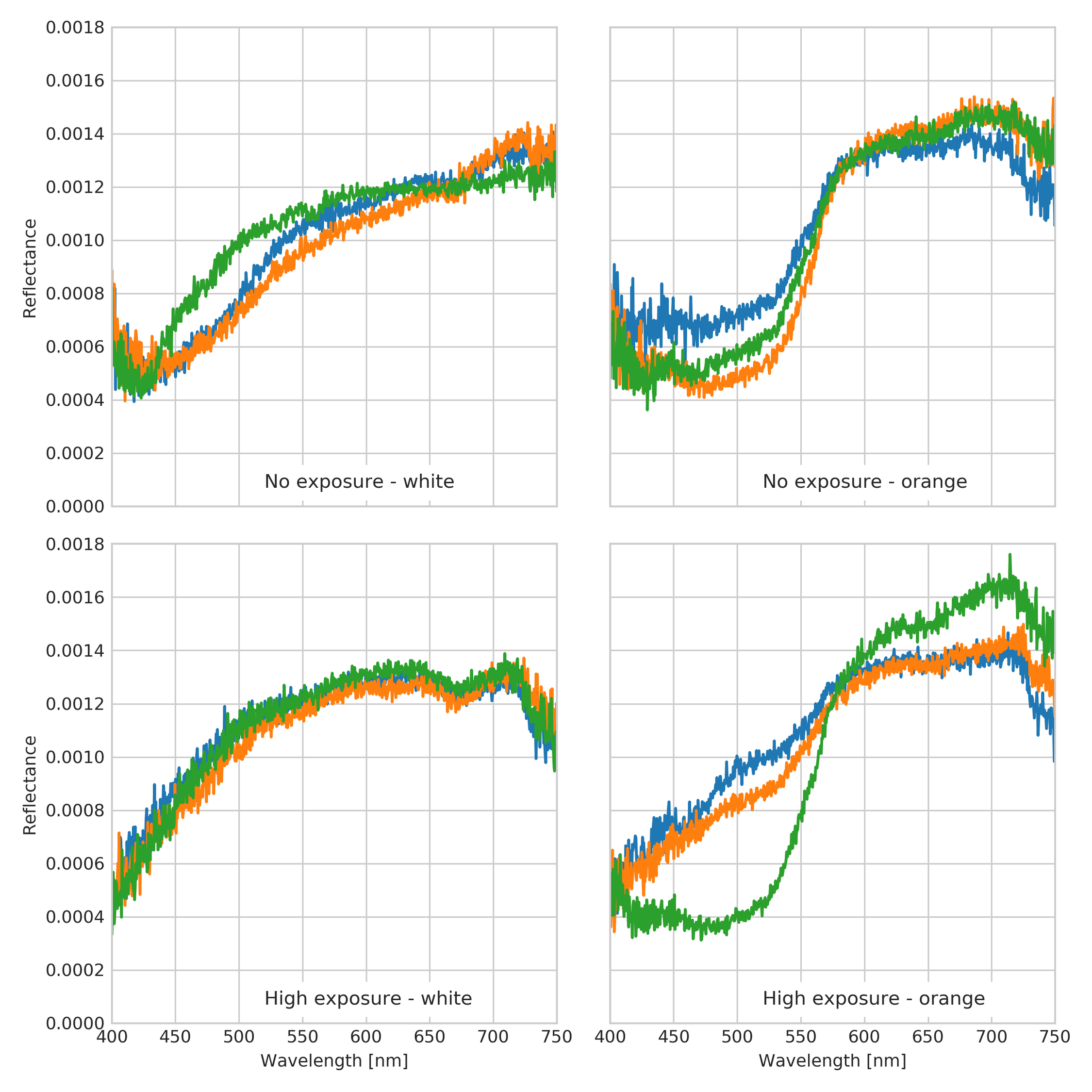

Supplement: S1 Fig — Reflectance spectra of white unexposed corals (top left), orange unexposed corals (top right), white high exposure corals (bottom left), and orange high exposure corals (bottom right). For each subfigure, all three spectra are taken from the same specimen. (TIF) [file pone.0209960.s003.tif]

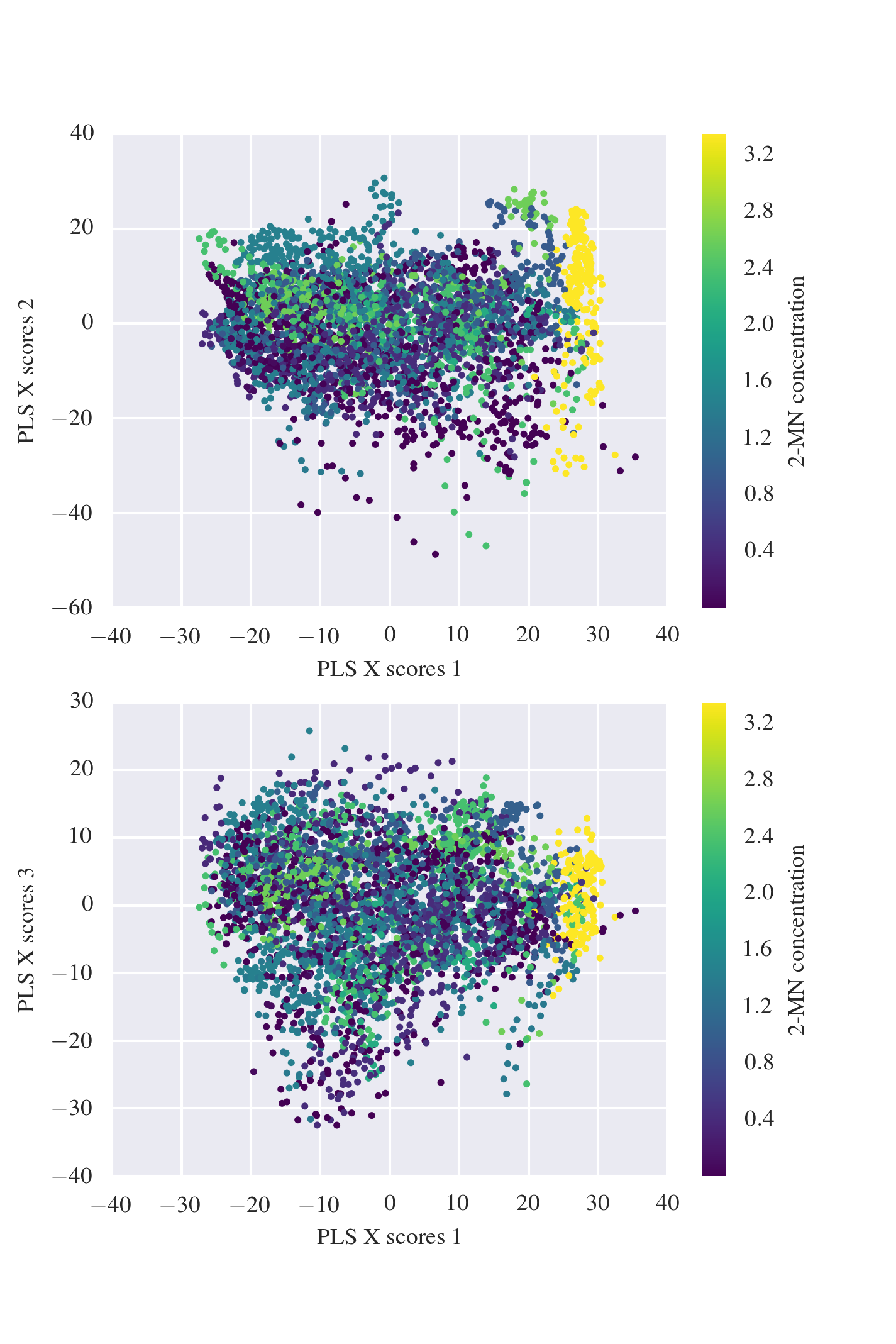

Supplement: S2 Fig — PLS X scores for orange corals are given in S2 Fig. The results correspond exactly to those presented in Fig 8, except that S2 Fig presents results for the orange color morph. (TIF) [file pone.0209960.s004.tif]

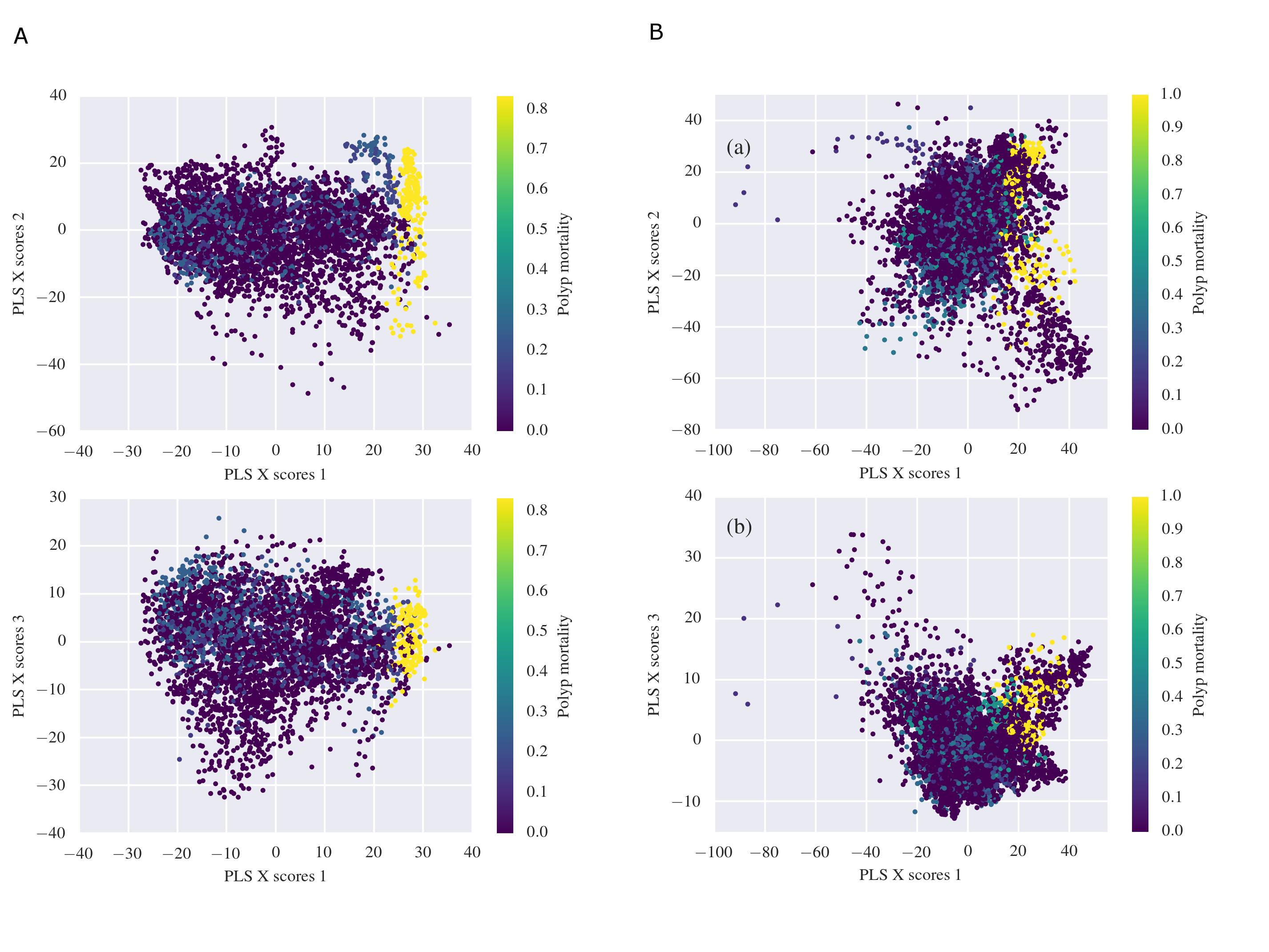

Supplement: S3 Fig — PLS X scores colored with the mortality variable are given for orange (A) and white (B) corals are given in S3 Fig. The results correspond exactly to those presented in Fig 8 and S2 Fig, except for the coloring of the dots. (TIF) [file pone.0209960.s005.tif]

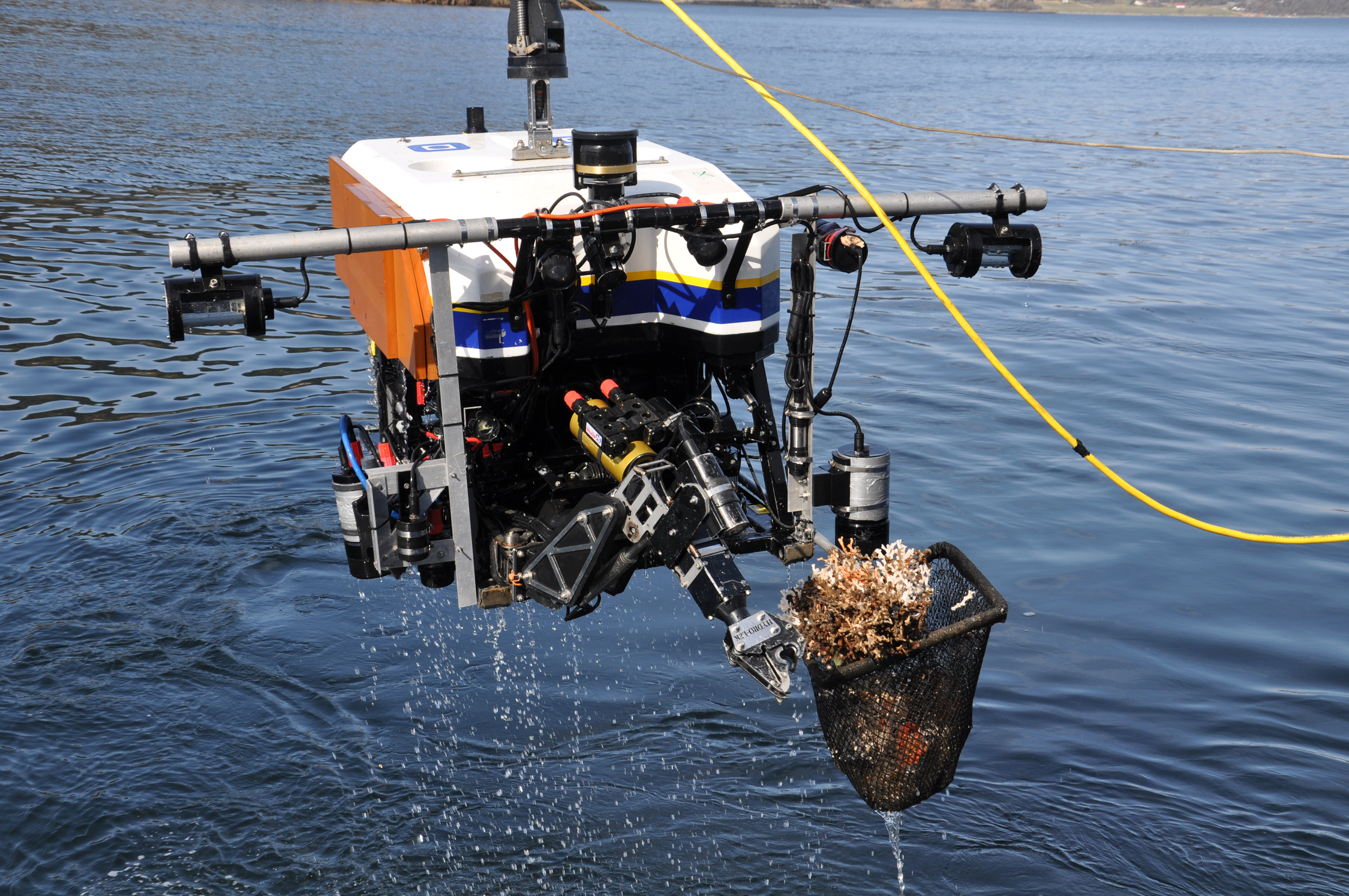

Supplement: S4 Fig — The ROV was maneuvered towards the rocky wall growing L. pertusa colonies, and the fish net was carefully poked towards the corals from below. This resulted in pieces of the coral colony breaking off and landing in the fish net. Precaution was taken to avoid any accidental damage of adjacent colonies. (TIF) [file pone.0209960.s006.tif]

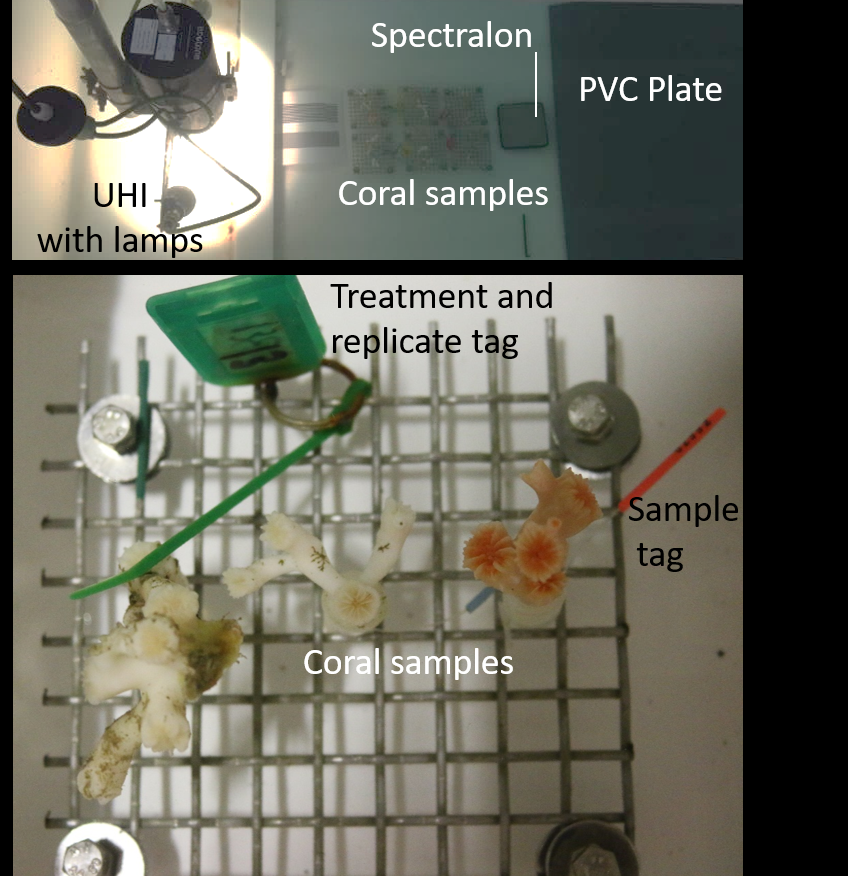

Supplement: S5 Fig — shows the experimental set-up for hyperspectral imaging. The photo corresponds to schematic sketch given in Fig 3. Experimental setup for UHI image acquisition. The upper image shows coral samples placed on grids at the bottom of a water filled tank. The UHI with lamps were attached to a scanning mechanism, and was set to image the tank bottom scenery while moving in a “push broom” fashion, keeping constant speed and distance to the tank bottom. A Spectralon reference plate and a PVC plate was used to correct for the inherent optical properties of the water. The lower photo details three coral samples attached to a steel grid. (TIF) [file pone.0209960.s007.tif]
